# Validation of the antibacterial effect of topically applied tranexamic acid using *in vitro* and *in vivo* models

**DOI:** 10.3389/fmicb.2024.1367884

**Published:** 2024-05-14

**Authors:** Antonio Benjumea, Marta Díaz-Navarro, Ángela Sai Gago-Campos, Andrés Visedo, Rama Hafian, Emilia Cercenado, Mar Sánchez-Somolinos, Patricia Muñoz, Javier Vaquero, Francisco Chana, María Guembe

**Affiliations:** ^1^Department of Orthopaedic Surgery and Traumatology, Hospital General Universitario Gregorio Marañón, Madrid, Spain; ^2^Department of Clinical Microbiology and Infectious Diseases, Hospital General Universitario Gregorio Marañón, Madrid, Spain; ^3^Instituto de Investigación Sanitaria Gregorio Marañón, Madrid, Spain; ^4^School of Biology, Universidad Complutense de Madrid, Madrid, Spain; ^5^Department of Biomedicine and Biotechnology, Universidad de Alcalá de Henares, Alcala de Henares, Spain; ^6^CIBER Enfermedades Respiratorias-CIBERES (CB06/06/0058), Madrid, Spain; ^7^School of Medicine, Universidad Complutense de Madrid, Madrid, Spain

**Keywords:** tranexamic acid, vancomycin, gentamicin, synergistic effect, reduction, MIC, biofilm, murine model

## Abstract

**Background:**

Several studies have shown that tranexamic acid (TXA), an antifibrinolytic, reduces postoperative infection rates. Recent *in vitro* research showed that TXA alone and in combination with vancomycin and gentamicin had a synergistic effect against some staphylococcal strains. In the present study, this synergistic effect was validated in samples from patients with staphylococcal periprosthetic infection (PPI) and in an *in vivo* model.

**Methods:**

We tested 19 clinical strains (5 *Staphylococcus aureus* and 14 coagulase-negative staphylococci [CoNS]) against 10 mg/ml TXA alone and in combination with serial dilutions of vancomycin and gentamicin. The standardized microtiter plate method was used. The minimal inhibitory concentration (MIC) were calculated using standard visualization of well turbidity. We also used an *S. aureus* (ATCC29213) murine subcranial PPI model to compare the synergistic effect of TXA and gentamicin with that of TXA or gentamicin alone after 4 days of monitoring. The mice were euthanized, and disks were removed for analysis of cfu/ml counts and cell viability rate. Biofilm structure of both *in vitro* and *in vivo* samples was also analyzed using scanning electron microscopy (SEM).

**Results:**

When TXA was combined with vancomycin or gentamicin, the MIC decreased in 30% of the strains studied. According to species, the MIC_50_ for vancomycin and gentamicin alone and in combination with TXA against *S. aureus* strains was the same. This was also the case for CoNS with vancomycin and its corresponding combination, whereas with gentamicin and TXA, a reduction in MIC_50_ was observed (2 dilutions). In addition, in the *in vivo* model, the mean (SD) log cfu/ml and cell viability rate obtained from the implant was lower in the group of mice treated with TXA and gentamicin than in those treated only with TXA or gentamicin. SEM images also corroborated our findings in strains in which the MIC was reduced, as well as the in the mice implants, with the area occupied by biofilm being greater in samples treated only with gentamicin or TXA than in those treated with TXA+gentamicin.

**Conclusion:**

We confirm that combining TXA with vancomycin or gentamicin exerts a synergistic effect. However, this only occurs in selected strains.

## Introduction

Periprosthetic infection (PPI) continues to be one of the main complications of prosthetic surgery, despite application of measures to prevent it during the perioperative period (Kapadia et al., 2016; Ratto et al., 2016). Biofilm formation favors the persistence of PPI and reduces the effect of antibiotics, thus hampering treatment. The most effective approach to treating PPI is preventing onset (Kapadia et al., 2016; Shoji and Chen, 2020).

Tranexamic acid (TXA) is used routinely in conventional surgery. It is currently indicated for hemostatic control of patients undergoing surgical interventions (Jennings et al., 2016; Waddell et al., 2016; Ockerman et al., 2021). Its mechanism of action is similar to that of lysine, which is capable of binding to plasminogen, preventing fibrinolysis, and, therefore, controlling hemorrhage (Jennings et al., 2016; Draxler et al., 2019).

TXA seems to be associated with a reduction in postoperative complications, including surgical wound infection. Based on the reported decrease in postoperative hematoma, some authors believe this is due to an indirect effect (Klement et al., 2020; Lacko et al., 2020; Sukeik et al., 2020; Yazdi et al., 2020). Other authors reported that TXA exerts a direct antibacterial effect on planktonic cells (Zhang et al., 2021; Benjumea et al., 2022b; Wang et al., 2022) and, moreover, that it produces a synergistic effect with antibiotics (Benjumea et al., 2022a). However, these results need to be validated.

The aim of our study was to assess the synergistic effect of TXA with vancomycin and gentamicin in clinical strains isolated from patients with staphylococcal PPI and to corroborate our findings in an *in vivo* model.

## Materials and methods

The study was carried out at a tertiary teaching Institution in Madrid, Spain.

### *In vitro* model

From December 2021 to March 2023, we prospectively collected 19 clinical strains (14 joint prosthesis, 2 tissue biopsy, and 3 osteosynthesis material) isolated from 17 patients with PPI.

Species distribution was as follows: *Staphylococcus aureus*, 5 (3 methicillin-susceptible and 2 methicillin-resistant); *Staphylococcus epidermidis*, 9; *Staphylococcus hominis*, 1; *Staphylococcus haemolyticus*, 1; and other coagulase-negative staphylococci (CoNS), 3.

#### Reduction in turbidity by TXA

We tested 10 mg/ml TXA (shown to be optimal) (Benjumea et al., 2022a,b; Wang et al., 2022) following CLSI guidelines using a microtiter plate (Clinical and Laboratory Standards Institute, 2018). The wells of a 96-well plate were inoculated with 50 μl of a suspension of 10^7^ cfu/ml of each microorganism in Müller-Hinton broth and treated with 50 μl of 20 mg/ml TXA (as the final dose in the well will be 10 mg/ml). Positive controls (bacterial suspension) were treated with 50 μl of sterile water, and negative controls (only Müller-Hinton broth) were treated with 50 μl of 20 mg/ml TXA. All the experiments were performed in triplicate. Plates were incubated at 37°C for 24 h.

We used a spectrophotometer (492 nm) to calculate the percentage reduction in turbidity for 10 mg/ml TXA and for positive control in each well.

#### MIC of vancomycin and gentamicin alone and in combination with TXA

We then tested the effect of 10 mg/ml TXA in combination with serial dilutions of vancomycin (Merck KGaA, Darmstadt, Germany) (from 0.03 mg/l to 16 mg/l) and gentamicin (Merck KGaA, Darmstadt, Germany) (from 0.06 mg/l to 32 mg/l), which were also tested separately in parallel.

The procedure was performed following CLSI guidelines (32nd edition, 2018) using a microtiter plate (Clinical and Laboratory Standards Institute, 2018). The wells of a 96-well plate were inoculated with 5 μl of a suspension of 10^7^ cfu/ml of each microorganism in Müller-Hinton broth and treated with the following: vancomycin (from 0.03 to 16 mg/L) alone (100 μl) and in combination (50 μl of twice the final dose in the well) with 50 μl of 20 mg/ml TXA (as the final dose in the well will be 10 mg/ml) and gentamicin (from 0.06 to 32 mg/l) alone (100 μl) and in combination (50 μl of twice the final dose in the well) with 20 mg/ml TXA. Positive controls (bacterial suspension) were treated with 100 μl of sterile water, and negative controls (only Müller-Hinton broth) were treated with 100 μl of each highest antibiotic concentration. All the experiments were performed in triplicate. Plates were incubated at 37°C for 24 h.

We defined the MIC of each antibiotic tested, alone or in combination with TXA, as the lowest concentration at which complete absence of well bacterial growth (compared with the positive control) was observed by the researcher (Clinical and Laboratory Standards Institute, 2018).

### *In vivo* model

We designed an *S. aureus* (ATCC29213) murine subcranial PPI model to compare the synergistic effect of 10 mg/ml TXA and gentamicin with that of TXA or gentamicin alone. We used 41 BALB/c mice (12-week-old), divided into 4 study groups: Group 1 (*n* = 10), control (no infection, polymethyl methacrylate [PMMA] without antibiotic or placebo administered with percutaneous saline); Group 2 (*n* = 12, including 2 additional mice from a preliminary pilot study), PMMA contaminated with bacteria and administration of percutaneous TXA; Group 3 (*n* = 10), PMMA with gentamicin 1.25% w/w contaminated with bacteria and administration of percutaneous TXA; and Group 4 (*n* = 9, 1 mouse died before surgery), PMMA with gentamicin 1.25% w/w contaminated with bacteria and administration of percutaneous saline. Two 5-mm bone cement disks (PMMA) per animal with or without gentamicin previously contaminated with *S. aureus* (1 × 10^7^ cfu) (controls only with media) were implanted in the skull. TXA or PBS (in controls) was administered subcutaneously (66 μl) after surgery. Four days later, the mice were euthanized with an overdose of anesthetics, and the PMMA disks were removed for analysis. For each animal, one of the disks was sonicated in 1 ml of PBS for 10 min, and the other was fixed with 1 ml of 2% glutaraldehyde for 24 h at 2–4°C for SEM analysis. The sonicate was divided to assess the cfu/ml counts expressed on a logarithmic scale by conventional culture on blood agar plates, and the cell viability rate was analyzed using flow cytometry (Gallios, Beckman Coulter, BioRad, Madrid, Spain). The flow cytometry results could only be analyzed in 11 out of the 31 samples (35.5%) because of alteration/degeneration during long-term storage (group 2, 4/12; group 3, 2/10; group 4, 5/9).

Samples of lung, brain, kidney, and blood (0.1 ml, by intracardiac puncture after euthanasia) were also collected to assess whether *S. aureus* infection was disseminated or whether other commensal microorganisms were present. Organs were homogenized with 1 ml of sterile saline, and 100 μl was cultured on blood and Brucella agar plates. Blood samples were directly inoculated into an aerobic blood culture bottle that was incubated in the automatized BACTEC System (BD Bactec™ FX) for 5 days at 37°C under continuous shaking. If the blood culture turned positive, 100 μl of the content was cultured on blood agar plates.

### Scanning electron microscopy

#### *In vitro* samples

To observe the structure of biofilms treated with 10 mg/ml TXA and antimicrobials (alone and in combination), we selected those clinical strains in which MIC had decreased in the *in vitro* experiments. The concentrations of vancomycin and gentamicin used were 32 mg/l and 64 mg/l, respectively. These antibiotic concentrations were chosen because they were 1 MIC higher than those shown by these species in a planktonic model in previous susceptibility studies (Benjumea et al., 2022b).

Biofilm was allowed to form on 12 mm diameter glass disks pre-treated with poly-L-lysine for 24 h at 37°C to promote initial bacterial adhesion. After this period, the disks were washed with PBS and transferred to a sterile 24-well plate. Then, 1 ml of each 0.5 McFarland bacterial suspension (corresponding to ~10^8^ cfu/ml) was added to the corresponding wells. After a 24-h incubation period at 37°C, the disks were washed with PBS to remove the planktonic bacteria. The biofilm formed on the disks was fixed with 300 μl of 2% glutaraldehyde for 24 h at 2–4°C.

#### *In vivo* samples

PMMA disks withdrawn from mice in the *in vivo* model were also analyzed.

#### Sample manipulation

Samples from both *in vitro* and *in vivo* experiments were dehydrated by subjecting them to increasing concentrations of ethanol (30%, 50%, 70%, 80%, and 90%) for 10 min and absolute ethanol for 20 min.

Differences in the structure and occupancy of the different biofilms from the *in vitro* and the *in vivo* experiments were analyzed using 2500x and 500x objectives, respectively (JEOL 6400 JSM).

### Data analysis

The effect of 10 mg/ml TXA alone against planktonic bacteria was expressed as a percentage reduction in well turbidity compared with the positive control.

The MIC of vancomycin and gentamicin alone and in combination with TXA was expressed as the geometric mean (MIC_50_) with the corresponding ranges.

Data were expressed by combining results from all species together and separately according to species (*Staphylococcus aureus, n* = 5; and *Staphylococcus epidermidis, n* = 14).

Quantitative variables are expressed as mean (SD) and comparisons between groups were performed using the median test. Significance was set at p < 0.05. Qualitative variables are expressed as no. (%) and comparisons between groups were performed using the chi-square test.

All tests were performed using SPSS Statistics for Windows, v.21.0 (IBM Corp, Armonk, New York, USA).

Data are stored in repository C.0001228 of the ISCIII.

### Ethics approval

For the *in vitro* study, the requirement of ethical approval was waived by Gregorio Marañón Local Ethics Committee on research involving medicinal products for the studies involving humans because the strains used were from clinical isolates without using patient data. The studies were conducted in accordance with the local legislation and institutional requirements. The ethics committee/institutional review board also waived the requirement of written informed consent for participation from the participants or the participants' legal guardians/next of kin because The strains used are from clinical isolates without using patient data.

For the *in vivo* model, the study was approved by Dirección General de Agricultura, Ganadería y Alimentación. Consejería de Medio Ambiente, Vivienda y Agricultura (PROEX no. 257.6/22). The study was conducted in accordance with the local legislation and institutional requirements.

## Results

### Reduction in turbidity by TXA

Overall, the mean (SD) percentage reduction in well turbidity for 10 mg/ml TXA was 4.5 (9.8) (*p* = 0.076). In particular, the mean (SD) percentage reduction in *S. aureus* and *S. epidermidis* strains was, respectively, 10.9 (15.5) (*p* = 0.416) and 2.2% (5.4) (*p* = 0.217) (Table 1).

**Table 1:** Mean (SD) absorbance and percentage reduction in well turbidity for 10 mg/ml TXA for each species.

**Strain**	Mean (SD) absorbance^*^of +C	Mean (SD) absorbance^*^of TXA	**Mean (SD) percentage reduction**	***p***
*S. aureus*	0.232 (0.059)	0.203 (0.740)	10.9 (15.5)	0.416
MSSA-13	0.179 (0.022)	0.139 (0.065)	23.7 (28.6)	
MSSA-14	0.216 (0.009)	0.166 (0.021)	14.4 (17.3)	
MSSA-17	0.182 (0.019)	0.148 (0.014)	8.4 (7.3)	
MRSA-34	0.261 (0.018)	0.277 (0.045)	0.0 (0.0)	
MRSA-35	0.323 (0.029)	0.285 (0.021)	8.1 (7.1)	
CoNS	0.159 (0.060)	0.185 (0.062)	2.2 (5.4)	0.217
*S. epidermidis*-15	0.110 (0.030)	0.210 (0.026)	0.0 (0.0)	
*S. epidermidis*-16	0.156 (0.027)	0.178 (0.022)	0.2 (0.3)	
*S. epidermidis*-18	0.205 (0.073)	0.189 (0.250)	8.5 (14.2)	
*S. epidermidis*-19	0.249 (0.514)	0.249 (0.068)	3.6 (6.2)	
*S. epidermidis*-26	0.178 (0.168)	0.195 (0.213)	0.0 (0.0)	
*S. hominis*-27	0.114 (0.061)	0.145 (0.013)	4.6 (7.9)	
CoNS-28	0.176 (0.019)	0.185 (0.043)	4.0 (6.9)	
*S. haemolyticus*-29	0.217 (0.033)	0.30 (0.011)	0.0 (0.0)	
CoNS-30	0.217 (0.172)	0.226 (0.028)	0.0 (0.0)	
*S. epidermidis*-31	0.129 (0.005)	0.185 (0.005)	0.0 (0.0)	
*S. epidermidis*-32	0.127 (0.025)	0.180 (0.050)	0.0 (0.0)	
*S. epidermidis*-33	0.043 (0.001)	0.048 (0.003)	0.0 (0.0)	
*S. epidermidis*-36	0.105 (0.035)	0.123 (0.037)	0.0 (0.0)	
*S. epidermidis*-37	0.198 (0.019)	0.177 (0.017)	10.4 (2.7)	
Overall	0.178 (0.068)	0.189 (0.065)	4.5 (9.8)	0.076

SD, standard deviation; +C, positive control; TXA, tranexamic acid; CoNS, coagulase-negative staphylococci.

^*^Absorbance was measured at 490 nm.

### MIC of vancomycin and gentamicin alone and in combination with TXA

**Figure 1:**
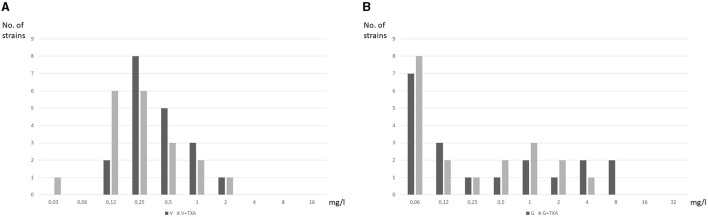
Distribution of strains according to MIC of vancomycin and gentamicin alone and in combination with TXA. **(A)** Vancomycin. **(B)** Gentamicin.

The distribution of strains according to the MIC of vancomycin and gentamicin alone and in combination with TXA is detailed in Figures 1A, B. In the combination of TXA with vancomycin in particular, an antibacterial effect was observed in 6 strains (31.5%), mostly CoNS (Figure 1A). The most notable reduction in MIC was observed in MRSA-35 (5 dilutions) and *S. epidermidis*-33 (3 dilutions). As for TXA combined with gentamicin, 6 strains (31.5%) other than those treated with vancomycin, in which MIC was reduced when gentamicin was combined with TXA (Figure 1B). The greatest reduction in MIC was observed in the *S. epidermidis*-16 and *S. epidermidis*-32 strains (2 dilutions both). In the remaining 4 strains, the MIC was only reduced by 1 dilution.

TXA-10 mg/ml reduced the MIC_50_ of gentamicin 2-fold only in CoNS: TXA10-G, < 0.06 mg/L vs. G, 0.25 mg/L (Table 2). No reduction was observed in the MIC_50_ of vancomycin.

**Table 2:** MIC_50_ (ranges) for vancomycin and gentamicin alone and in combination with TXA.

**Treatment**	***S. aureus* MIC_50_ (range) mg/l**	**MIC_50_ fold reductions (no.)**	**CoNS MIC_50_ (range) mg/l**	**MIC_50_ fold reductions (no.)**	**Overall MIC_50_ (range) mg/l**	**MIC_50_ fold reductions (no.)**
Vancomycin	0.25 (0.25–1)	0	0.25 (0.12–2)	0	0.25 (0.12–2)	0
Vancomycin+10 mg/ml TXA	0.25 (< 0.03–0.5)		0,25 (0,12–2)		0.25 (< 0.03–2)	
Gentamicin	0.12 (< 0.06–2)	0	0.25 (< 0.06–8)	2	0.12 (< 0.06–8)	0
Gentamicin+10 mg/ml TXA	0.12 (< 0.06–1)		< 0.06 (< 0.06–8)		0.12 (< 0.06–8)	

TXA, tranexamic acid; MIC, minimal inhibitory concentration; no., number; CoNS, coagulase-negative staphylococci.

### Effect of TXA and gentamicin on PMMA disks in the *in vivo* murine model

The mean (SD) log cfu/ml obtained from the sonicate of the implant was lower in the group of mice treated with TXA and gentamicin (group 3) (0.853 [1.229]) than in those treated only with TXA (group 2) (6.181 [1.968], *p* < 0.001; 86.2% reduction) or gentamicin (group 4) (1.818 [2.192], *p* = 0.246; 53.1% reduction). Similar results were obtained for cell viability rates, where the mean (SD) percentage of viable cells obtained from the sonicate was lower in group 3 (2.25 [1.202]) than in group 2 (54.63 [5.014], *p* < 0.001; 95.9% reduction) and group 4 (3.26 [5.716], *p* = 0.824; 31.0% reduction) (Table 3). Crude log cfu/ml values and the cell viability rate are provided in Supplementary File 1.

**Table 3:** Mean (SD) log cfu/ml counts, cell viability rate, and colonized samples of *Staphylococcus aureus* from the murine study groups.

**Study group**	**Mean (SD) log cfu/ml**	Mean (SD) % cell viability^*^	**Brain colonization, *N* (%)**	**Lung colonization, *N* (%)**	**Bacteremia, *N* (%)**
2 (10 mg/ml TXA)^a, b^	6.181 (1.968)	54.63 (5.014)	8 (66.7)	3 (25.0)	0 (0.0)
3 (10 mg/ml TXA + gentamicin 1.25% w/w)^c^	0.853 (1.229)	2.25 (1.202)	2 (20.0)	2 (20.0)	0 (0.0)
4 (gentamicin 1.25% w/w)	1.818 (2.192)	3.26 (5.716)	4 (44.4)	2 (22.2)	1 (11.1)

SD, standard deviation; cfu, colony-forming units; TXA, tranexamic acid.

^*^Cell viability could only be assessed at 11 out of the 31 samples.

^a^p value between groups 2 and 3: log cfu/ml, p < 0.001; cell viability, p < 0.001; brain colonization, p = 0.043; lung colonization, p = 1.000; bacteremia, p = not applicable; ^b^p value between groups 2 and 4: log cfu/ml, p < 0.001; cell viability, p < 0.001; brain colonization, p = 0.396; lung colonization, p = 1.000; bacteremia, p = 0.409; ^c^p value between groups 3 and 4: log cfu/ml, p = 0.246; cell viability, p = 0.824; brain colonization, p = 0.350; lung colonization, p = 1.000; bacteremia, p = 0.450.

**Figure 2:**
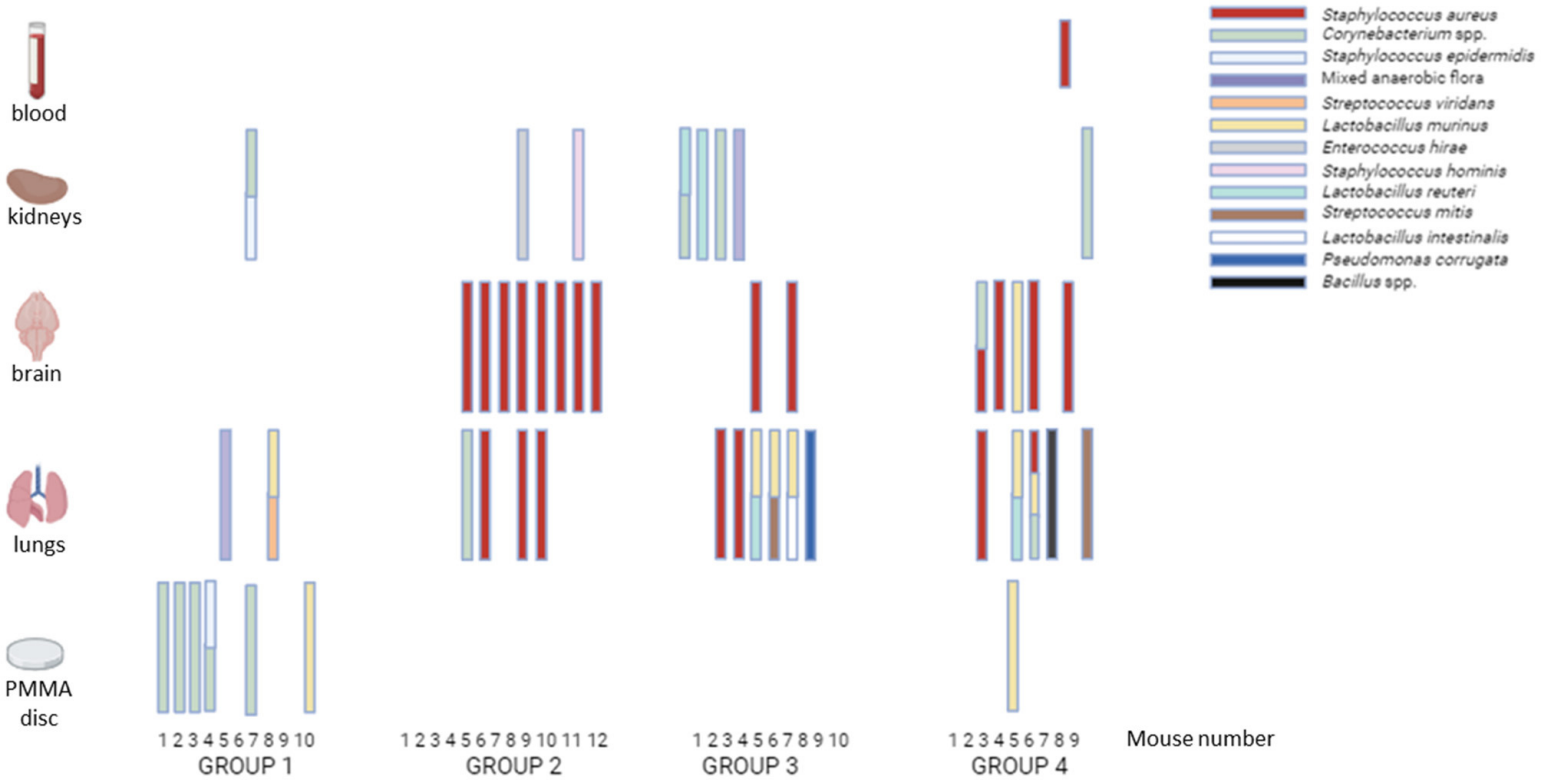
Microorganisms isolated from samples obtained from the *in vivo* study. Group 1: negative control; Group 2: TXA; Group 3: TXA + gentamicin; Group 4: gentamicin. Samples of lung, brain, kidney, and blood (0.1 ml, by intracardiac puncture after euthanasia) were collected and homogenized with 1 ml of sterile saline. Then, 100 μl was cultured on blood and Brucella agar plates. Blood samples were directly inoculated into an aerobic blood culture bottle that was incubated in the automatized BACTEC System (BD Bactec™ FX) for 5 days at 37°C under continuous shaking. If the blood culture turned positive, 100 μl of the content was cultured on blood agar plates.

The lowest *S. aureus* colonization rate in murine samples was found in group 3 (TXA + gentamicin), as follows: lungs, 20.0%; brain, 20.0%; and blood, 0.0%. In addition, the difference in the *S. aureus* colonization rate in brain samples between groups 2 (TXA) and 3 was statistically significant (66.7% vs. 20.0%, *p* = 0.043) (Table 3, Figure 2). Only 1 mouse from group 4 developed bacteremia (*S. aureus* grew in blood culture).

As cell viability could only be assessed in 11 of the 31 samples, we observed the viable but non-culturable phenomenon in 5 of them (45.5%).

Microorganisms (both *S. aureus* and commensal flora) isolated from samples of each group are detailed in Figure 2.

### Biofilm structure analysis by scanning electron microscopy

**Figure 3:**
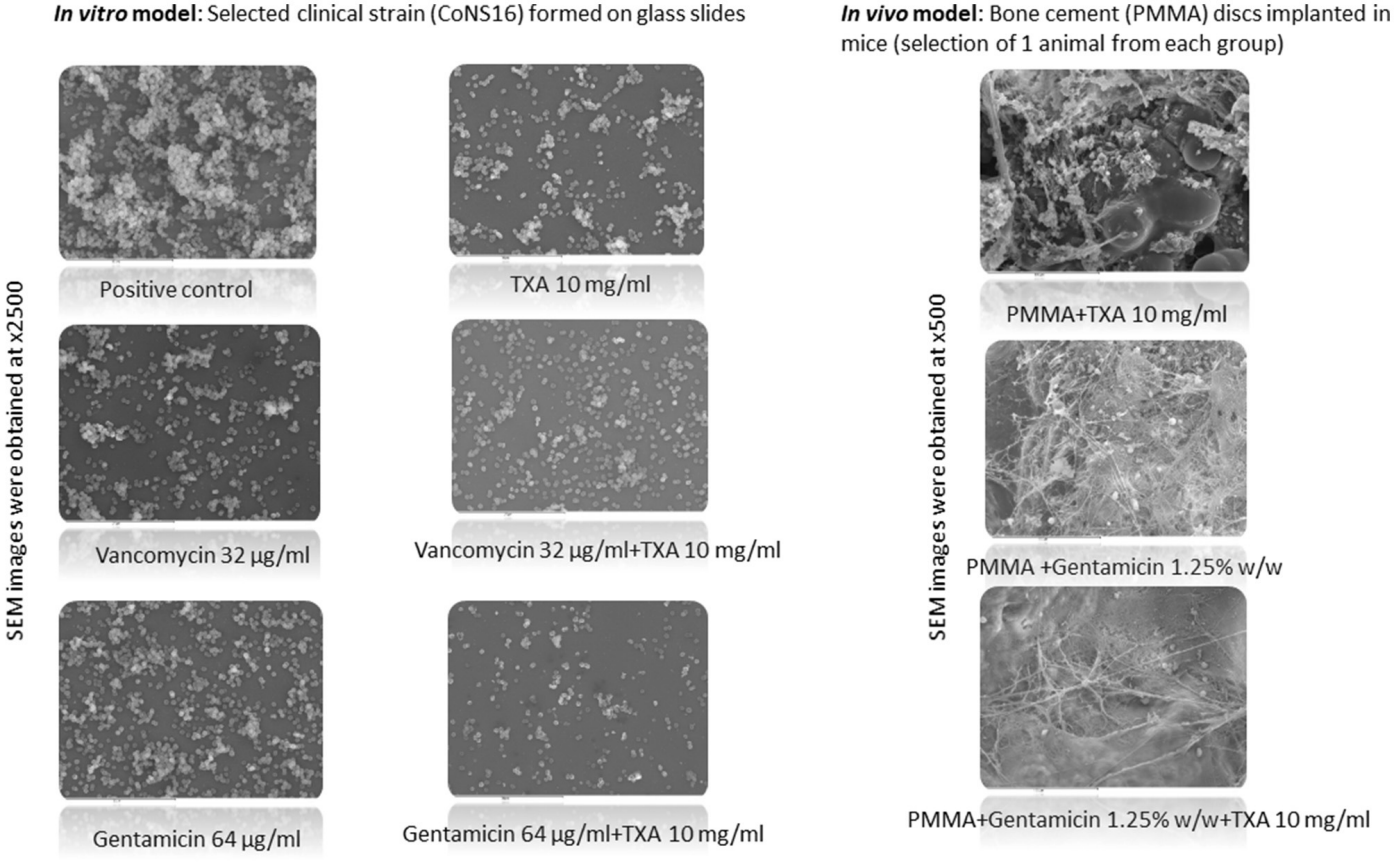
SEM images of a selected clinical strain and PMMA disks implanted in mice according to the different treatment groups. PMMA, polymethylmethacrylate; TXA, tranexamic acid; SEM, scanning electron microscopy. For CoNS16 strains, the vancomycin and gentamicin concentration used was 32 mg/l and 64 mg/l, respectively. Samples from both *in vitro* and *in vivo* experiments were dehydrated by subjecting them to increasing concentrations of ethanol (30%, 50%, 70%, 80%, and 90%) for 10 min and absolute ethanol for 20 min. Differences in the structure and occupancy of the different biofilms from the *in vitro* and the *in vivo* experiments were analyzed using 2500x and 500x objectives, respectively (JEOL 6400 JSM).

As can be seen in the images taken by scanning electron microscopy (SEM), a lower number of bacteria within the biofilm is observed on glass disks after being treated *in vitro* with 10 mg/ml TXA compared with the positive control. This effect is even more pronounced when biofilms were treated *in vitro* with gentamicin in combination with 10 mg/ml TXA. Similar results were obtained when analyzing the PMMA disc containing gentamicin 1.25% w/w followed by locally administered TXA 10 mg/ml in mice (Figure 3).

## Discussion

Our *in vivo* model corroborates our previous findings regarding the antimicrobial effect of TXA in PPI (Benjumea et al., 2022a). However, when we tested *in vitro* TXA against a large collection of clinical strains, we were able to demonstrate its synergistic effect in only 30% of them.

TXA has been associated with a decrease in the incidence of surgical wound complications, including PPI, in clinical studies (Klement et al., 2020; Lacko et al., 2020; Yazdi et al., 2020). However, the main mechanism of action through which the drug acts has not yet been established. It has been hypothesized that TXA could have a direct or indirect effect on the growth of microorganisms, and while we previously demonstrated the direct antibacterial effect of TXA (Benjumea et al., 2022a,b), some authors support an indirect effect by immunomodulation, which favors the host immune defense mechanisms against the pathogens that cause the infection. In addition, other studies support a direct hemostatic mechanism, which reduces the frequency of postoperative hematoma and thus the possibility of infectious complications (Draxler et al., 2019; Lacko et al., 2020; Sukeik et al., 2020; Yazdi et al., 2020).

In a previous study, we demonstrated the synergistic effect of TXA with vancomycin and gentamicin, which are the most widely used, locally applied antibiotics in orthopedic surgery (Benjumea et al., 2022a). However, in the present study, we decided to analyze the synergistic effect of 10 mg/ml TXA with these antibiotics against a large collection of clinical strains isolated from patients with musculoskeletal infection. We used a 10-mg/ml concentration for a number of reasons: first, because we had previously demonstrated that it had a direct antibacterial effect on planktonic cells and that its effect was not dose-dependent (Benjumea et al., 2022a; Wang et al., 2022); second, because it has been shown that ≥20 mg/ml can cause cellular toxicity (to chondral cells, epithelial cells, and fibroblasts), with the result that wound healing capacity could be compromised at higher doses (Parker et al., 2018; Eikebrokk et al., 2019; Bolam et al., 2021); and, third, because 10 mg/ml is the most widely used concentration in clinical studies (Sun et al., 2017; Montroy et al., 2018; Masaryk et al., 2022; Bi et al., 2023). Our analysis showed an overall reduction in turbidity of 10.9% for *S. aureus* and 2.2% for *S. epidermidis* strains when treated with TXA alone. However, in specific strains, this reduction ranged between 10% and 23%. Therefore, we found that the effect of TXA may be strain-dependent.

Furthermore, with the aim of assessing the synergistic effect in an *in vivo* model, where the environment could affect the previous *in vitro* results, we created a murine PPI model, which, to our knowledge, is the first to assess the synergistic effect of TXA with antibiotics. Only 2 previous studies have been based on *in vivo* models testing TXA, although neither of them used other compounds in combination (Zhang et al., 2021; Wang et al., 2022). We combined 10 mg/ml TXA and gentamicin-loaded bone cement (PMMA) in an *S. aureus* murine PPI model and demonstrated a synergistic effect (86.2% decrease in bacterial growth, compared with the group treated with TXA alone, as observed in the SEM images). In the group of mice in which only gentamicin-loaded PMMA was used, this reduction in bacterial growth reached 53.1%, corresponding to an approximately 35% reduced antibacterial effect. Although we only tested an ATCC *S. aureus* strain in the *in vivo* model, which already showed good results in the *in vitro* experiments, additional factors, such as the host's immune factors, also positively affected the synergistic effect. As hypothesized by Wang et al. (2022), TXA affects the expression of polysaccharides and proteins that favor the formation of bacterial aggregates, making biofilm formation difficult. This probably accounts for the effect of TXA that would make bacteria more susceptible to the action of both phagocytic cells and the antibiotic.

In addition, we found that the incidence of colonized brain samples was higher (66.7%) in the group treated with TXA only than in the group treated with TXA and gentamicin (20.0%), although no differences were observed with respect to the group treated with gentamicin only (20.0%).

Regarding the potential risks of TXA, including thromboembolic disease, it has not been demonstrated that the combination of TXA with locally applied antibiotics (joints) causes adverse effects. In orthopedic surgery, the use of antibiotic-loaded cement is a very widespread practice. Similarly, TXA is widely used topically and systemically, and no increase in the incidence of adverse effects has been described in any of its applications (Franchini et al., 2018; Montroy et al., 2018; Oliva-Moya et al., 2022).

One of the main limitations of our study is that our findings suggests that TXA effect is strain-dependent, so they cannot be applied universally across all staphylococcal strains encountered in clinical practice. In addition, as it was a prospective study, we included a small number of clinical strains. Based on this, future clinical studies assessing the use of TXA directly in the clinical scenario and including other antibiotics commonly used in the treatment of staphylococcal infections are required. There is also needed to better elucidate the molecular mechanisms underlying the observed synergistic effect of TXA and whether its addition to PMMA would affect the adhesion of bone cement.

In summary, our study is the first to analyze the synergistic effect of TXA and locally applied gentamicin on the growth of microorganisms in an *in vivo* model. We also demonstrated that the *in vitro* synergistic effect of TXA with gentamicin and vancomycin is strain-dependent. The main limitation of the study is that we only tested a single strain in the murine model.

## Conclusion

Our results showed that the ability of TXA to reduce bacterial growth is based on a direct anti-aggregative effect on biofilm formation, thus reducing the extracellular matrix, which enables antimicrobials to better reach bacteria. This may explain the synergism between 10 mg/ml TXA and vancomycin/gentamicin. However, this effect is not always observed, as it is strain-dependent.

## Data availability statement

The raw data supporting the conclusions of this article will be made available by the authors, without undue reservation.

## Ethics statement

The requirement of ethical approval was waived by Gregorio Marañón Local Ethics Committee on research involving medicinal products for the studies involving humans because the strains used are from clinical isolates without using patient data. The studies were conducted in accordance with the local legislation and institutional requirements. The Ethics Committee/institutional review board also waived the requirement of written informed consent for participation from the participants or the participants' legal guardians/next of kin because the strains used are from clinical isolates without using patient data. The animal study was approved by Dirección General de Agricultura, Ganadería y Alimentación. Consejería de Medio Ambiente, Vivienda y Agricultura (PROEX no. 257.6/22). The study was conducted in accordance with the local legislation and institutional requirements.

## Author contributions

AB: Conceptualization, Data curation, Investigation, Methodology, Writing—original draft. MD-N: Data curation, Formal analysis, Investigation, Methodology, Writing—original draft. ÁG-C: Investigation, Methodology, Writing—original draft. AV: Investigation, Methodology, Writing—original draft. RH: Investigation, Methodology, Writing—original draft. EC: Conceptualization, Supervision, Writing—review & editing. MS-S: Conceptualization, Investigation, Supervision, Writing—review & editing. PM: Supervision, Validation, Writing—review & editing. JV: Conceptualization, Supervision, Validation, Writing—review & editing. FC: Conceptualization, Supervision, Validation, Writing—review & editing. MG: Funding acquisition, Project administration, Resources, Supervision, Validation, Writing—review & editing.
