# Supplementary material for: Validation of the antibacterial effect of topically applied tranexamic acid using in vitro and in vivo models
Source: Front Microbiol. 2024 May 14;15:1367884. doi: 10.3389/fmicb.2024.1367884 (PMC11130467; doi:10.3389/fmicb.2024.1367884)
Supplement: Supplementary file 1 [file Table_1.DOCX]

**Supplemental file 1. Colony forming units and percentage cell viability of *Staphylococcus aureus* from each animal**

| **Animal no.** | **Group** | **cfu/ml** | **log cfu/ml** | **% cell viability (11/31)** |
| --- | --- | --- | --- | --- |
| **1** | **2 (10 mg/ml TXA)** | 14,000,000 | 7.15 |  |
| **2** |  | 9,400,000 | 6.97 |  |
| **3** |  | 9,600,000 | 6.98 |  |
| **4** |  | 9,600,000 | 6,98 |  |
| **5** |  | 3,200,000 | 6.51 | 52.7 |
| **6** |  | 5,200,000 | 6.72 | 48.5 |
| **7** |  | 4,000,000 | 6.60 | 57.7 |
| **8** |  | 4,000,000 | 6.60 | 59.6 |
| **9** |  | 4,200,000 | 6.62 |  |
| **10** |  | 1,210,000 | 6.08 |  |
| **11** |  | 0 | 0.00 |  |
| **12** |  | 9,200,000 | 6.96 |  |
| **1** | **3 (10 mg/ml TXA + gentamicin 1.25% w/w)** | 170 | 2.23 |  |
| **2** |  | 0 | 0.00 | 1.4 |
| **3** |  | 100 | 2.00 |  |
| **4** |  | 0 | 0.00 | 3.1 |
| **5** |  | 10 | 1.00 |  |
| **6** |  | 0 | 0.00 |  |
| **7** |  | 2,000 | 3.30 |  |
| **8** |  | 0 | 0.00 |  |
| **9** |  | 0 | 0.00 |  |
| **10** |  | 0 | 0.00 |  |
| **1** | **4 (gentamicin 1.25% w/w)** | 0 | 0.00 | 2.0 |
| **2** |  | 0 | 0.00 | 13.4 |
| **3** |  | 0 | 0.00 |  |
| **4** |  | 30,000 | 4.48 |  |
| **5** |  | 0 | 0.00 | 0.3 |
| **6** |  | 60,000 | 4.78 | 0.3 |
| **7** |  | 0 | 0.00 |  |
| **8** |  | 5,000 | 3,70 |  |
| **9** |  | 2,520 | 3.40 | 0.3 |

**cfu**, colony forming units; **TXA**, tranexamic acid.
